# Diagnosis of *Enterococcus faecalis* meningitis associated with long-term cerebrospinal fluid rhinorrhoea using metagenomics next-generation sequencing: a case report

**DOI:** 10.1186/s12879-021-06797-y

**Published:** 2021-10-26

**Authors:** Xiaobo Zhang, Chao Jiang, Chaojun Zhou

**Affiliations:** Department of Neurology, First People’s Hospital of Changde City, Changde, Hunan China

**Keywords:** Meningitis, *Enterococcus faecalis*, Next-generation sequencing, Cerebrospinal fluid

## Abstract

**Background:**

*Enterococcus faecalis* (*E. faecalis*) meningitis is a rare disease, and most of its occurrences are of post-operative origin. Its rapid diagnosis is critical for effective clinical management. Currently, the diagnosis is focused on cerebrospinal fluid (CSF) culture, but this is quite limited. By comparison, metagenomic next-generation sequencing (mNGS) can overcome the deficiencies of conventional diagnostic approaches. To our knowledge, mNGS analysis of the CSF in the diagnosis of *E. faecalis* meningitis has been not reported.

**Case presentation:**

We report the case of *E. faecalis* meningitis in a 70-year-old female patient without a preceding history of head injury or surgery, but with an occult sphenoid sinus bone defect. *Enterococcus faecalis* meningitis was diagnosed using mNGS of CSF, and she recovered satisfactorily following treatment with appropriate antibiotics and surgical repair of the skull bone defect.

**Conclusions:**

Non-post-traumatic or post-surgical *E. faecalis* meningitis can occur in the presence of occult defects in the cranium, and mNGS technology could be helpful in diagnosis in the absence of a positive CSF culture.

## Background

*Enterococcus faecalis* is a common species of enterococcus, belonging to Gram-positive cocci [1]. Cases of *E. faecalis* meningitis are rarely reported, with an incidence of 0.3–4% of bacterial meningitis [2]. However, it causes a severe purulent meningitis, which occurs mostly in patients with brain trauma or following surgery [3].

In addition, congenital defects can contribute to the development of meningitis [4]. Cerebrospinal fluid rhinorrhea or otorrhea is generally among the presenting symptoms or even a leading symptom in cases of meningitis associated with skull defects of traumatic or neurosurgical origin [5]. However, reports of *E. faecalis* meningitis cases unrelated to trauma or surgery are rare.

And, while, conventional diagnostic methods such as culture still have a place in the evaluation of clinical specimens, mNGS, as an emerging detection technology, can improve diagnostic accuracy in the presence of negative traditional culture methods. It has a higher positive rate than traditional culture methods, and it can identify novel or rare pathogens within 1–2 days [6]. Here, we report the case of *E. faecalis* meningitis diagnosed using mNGS analysis.

## Case presentation

The patient, a 70-year-old woman was referred to our hospital in June 2020 because of sudden headache, fever and unconsciousness for 7 h. She had no history of treatment or nose picking before admission. She was a farmer who raised poultry and had a history of renal calculi but was without clinical attacks. Physical examination showed signs of meningeal irritation. On the day of admission, cranial CT showed no abnormality. Routine blood testing showed increased white blood cell count of 14.28 × 10^9^/L (normal range 3.5 to 9.5 × 10^9^/L), with 85.6% neutrophils, and an elevated C-reactive protein of 32.87 mg/L (normal range < 10 mg/L). Together with clinical presentation and physical examination, a clinical diagnosis of meningitis was made and a lumbar puncture done, and CSF samples sent for ‘routine analysis’ and mNGS. The sample for the latter was sent to IngeniGen XunMinKang Biotechnology Inc. A blood culture was also done.

Analysis of the CSF (Table 1) confirmed the diagnosis of meningitis, but CSF staining, including staining for fungi, Gram staining and staining for acid fast bacilli, was negative.

**Table 1:** Baseline and subsequent cerebrospinal fluid parameters in a 70 years old lady with *E. faecalis* meningitis

CSF parameter	On the first day	On the 3rd day	On the 8th day
Appearance	Turbid	Turbid	Limpid
Pressure (cmH_2_0)	300	200	160
Erythrocyte count (/mm^3^)	340	130	10
WBC count (/mm^3^) (normal range, 0–10)	4950	760	31
WBC distribution (N/L)	80/20	70/30	80/20
Protein (mg/L) (normal range, 150–450)	4025	1738	850
CSF glucose (mmol/L) (normal range, 2.5–4.5)	2.48	2.36	2.0
Plasma glucose (mmol/L)	9.4	8.6	4.5
Gram stain	Normal	Normal	Normal

*WBC* white blood cell, *CSF* cerebrospinal fluid, *L* lymphocytes, *N* neutrophils

**Fig. 1:**
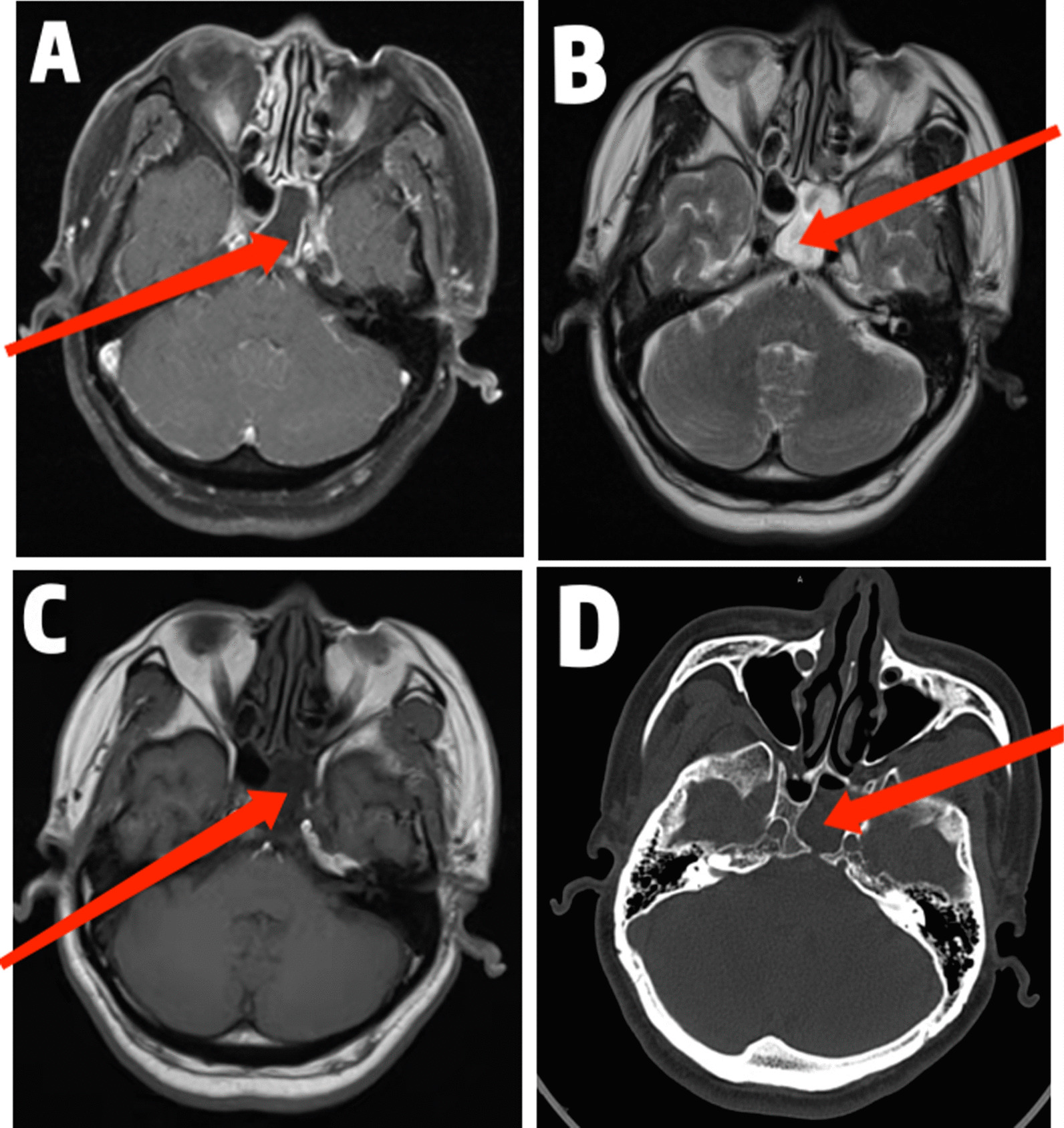
Imaging changes of the patient’s brain. MRI (**A**–**C**) and CT (**D**) indicates sphenoid sinus effusion as shown by the arrows

On the second day following admission, magnetic resonance imaging of the head revealed extensive enhancement of the meninges and sphenoid sinus effusion (Fig. 1A–C). The procalcitonin level was elevated to 1.52 ng/mL (normal range < 0.5 ng/mL). Coagulation function, hepatitis B surface antigen, syphilis antibody, HIV antibody, and hepatitis C antibody were in normal range. Detection of tuberculosis antibody, tumor markers of serum, and virus-related antibodies were also negative.

**Fig. 2:**
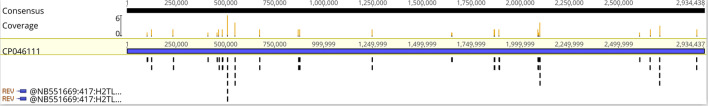
mNGS results of pathogen identification: mNGS analysis of the CSF confirmed the presence of *E. faecalis* with 36 sequences

On the third day following admission, repeated CSF examination (Table 1) showed persistence of the meningitis, but routine culture of CSF and CSF stain once again showed negative findings. However, by the fourth day after admission, the results of mNGS analysis of the CSF taken on admission showed the presence of *E. faecalis* (Fig. 2). No other pathogens were detected. Treatment with latamoxef sodium from the day of admission resulted in a gradual resolution of fever and improvement in the level of consciousness.

Five days after the onset of the disease, when she was now fully conscious, she gave a history of persistent rhinorrhea of four months before admission, and a 3-dimensional cranial CT imaging (Fig. 1D) confirmed sphenoid sinus effusion for which a neurosurgeon was consulted for further evaluation and management.

On the 8 day after admission, the patient was transferred to the Neurosurgery Ward and underwent repair of sphenoidal bone defect.

Another lumbar puncture on the 13th day following admission revealed that the CSF indicators were significantly improved (Table 1). She was released from the hospital after approximately 4 weeks of admission without any complications.

We followed up the patient for 6 months, but she refused to come back for further review as her physical condition was good without meningitis symptoms and CSF rhinorrhoea.

## Discussion and conclusion

*Enterococcus faecalis* was not previously considered pathogenic, but it has rapidly become the most prevalent nosocomial pathogen in recent years [1]. Most *E. faecalis* infections are endogenous, but CNS infections are relatively rare being only generally observed in adults secondary to neurosurgery, and in children with congenital malformations of the central nervous system [7].

The symptoms of *E. faecalis* meningitis are similar to those of other bacterial meningitis [8]. The patient in this report exhibited fever, severe headache, and loss of consciousness. Physical examination showed positive meningeal irritation signs, and laboratory tests of CSF indicated the characteristics of purulent meningitis. Traditional CSF culture and serological testing were all negative, but mNGS identified *E. faecalis* in CSF. This case started with manifestations of CNS infection, but a detailed inquiry revealed that the patient had a history of CSF rhinorrhoea. The cause of CSF rhinorrhoea was confirmed as sphenoid sinus bone defect through surgical exploration. The route of infection with *E. faecalis* in the patient may be endogenous through the CSF rhinorrhea. However, such a CSF leak may not always be overt or clinical, and radiological examinations may even fail to demonstrate a leak [9]. We also need to acknowledge the possibility of a truly exogenous infection in view of the patient’s occupation. Therefore, in our opinion, when an adult presents with purulent meningitis due to uncommon or rare bacterial pathogens, an occult defect or malformation of the CNS should be thoroughly sought.

In the diagnosis of meningitis, the traditional microbiological tests may yield negative results because of prior antibiotic treatment or the low number of bacteria in the CSF. However, these factors are not specific to the isolation of *E. fecalis*, but could be applicable generally to the isolation by culture of any bacterial pathogen [10]. To improve the diagnostic yield in our case, we used mNGS [6]. The detection of *E. faecalis* in CSF by next-generation sequencing has not been published previously, but it has comparative advantages. In addition, mNGS is appropriate for various specimen types, including peripheral blood, CSF, tissue, sputum, and bronchoalveolar lavage [11]. Many reports [12–16] show the strong potential of mNGS in infectious disease diagnostics compared with traditional methods.

In conclusion, this case highlights the place of next-generation sequencing in identification of the etiologic agent of meningitis. *E. faecalis* meningitis can occur in the absence of trauma or surgery in patients with preexisting anatomic cranial bone defects.

## Data Availability

The data that support the findings of the current study are available from the corresponding author upon reasonable request.

## Abbreviations

CSF: Cerebrospinal fluid
mNGS: Metagenomic next-generation sequencing
CNS: Central Nervous System
Fig: Figure
*E. faecalis*: *Enterococcus faecalis*
